# The Evolution of Pediatric Disease—A Moving Target in Public Health

**DOI:** 10.3390/diseases5030018

**Published:** 2017-08-31

**Authors:** Mark A. Brown

**Affiliations:** 1 Department of Clinical Sciences, Colorado State University, Fort Collins, CO 80523, USA; Mark.Brown@colostate.edu; Tel.: +01-970-491-5782; 2 Epidemiology Section, Colorado School of Public Health, Fort Collins, CO 80523, USA; 3 Cell and Molecular Biology Program, Colorado State University, Fort Collins, CO 80523, USA; 4 Department of Ethnic Studies, Colorado State University, Fort Collins, CO 80523, USA

**Keywords:** pediatric, emerging infectious disease, childhood vaccination

## Abstract

There is a growing threat in the re-emergence of diseases that impact pediatric demographics. While major strides have been made in the field of childhood cancers, there are still more questions than answers. In addition, public resistance to recommended practices related to childhood vaccinations fueled by misinformation has allowed infectious diseases to resurface in developed nations. Meanwhile, climate change and other destabilizing factors are shifting vector populations and driving the emergence of new diseases. Herein we call upon the community of human health researchers to confront the evolving specter of pediatric disease.

## 1. Introduction

**Figure 1:**
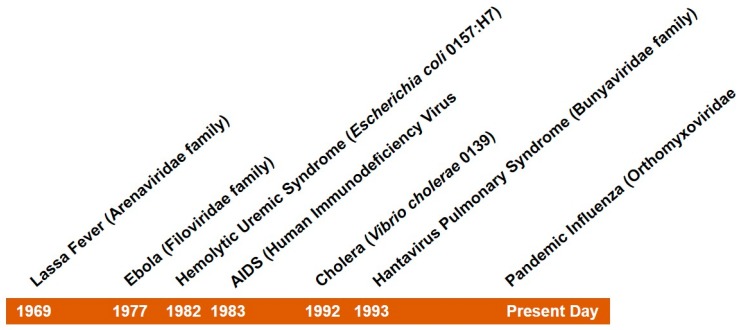
Emerging infectious diseases of the past fifty years.

The idea that deadly infectious diseases have been all but eradicated has receded with the emergence of new threats over recent decades such as HIV and hanta virus pulmonary syndrome [1,2]. Figure 1 provides examples of emerging infectious diseases of the past fifty years. Likewise, the failure of parents to follow recommended vaccination guidelines as a result of misguided fear related to the safety of vaccines has resulted in the re-emergence of previously well controlled childhood illnesses such as pertussis [3] and measles [4]. Worldwide, infectious diseases remain a leading cause of death among children. 

**Figure 2:**
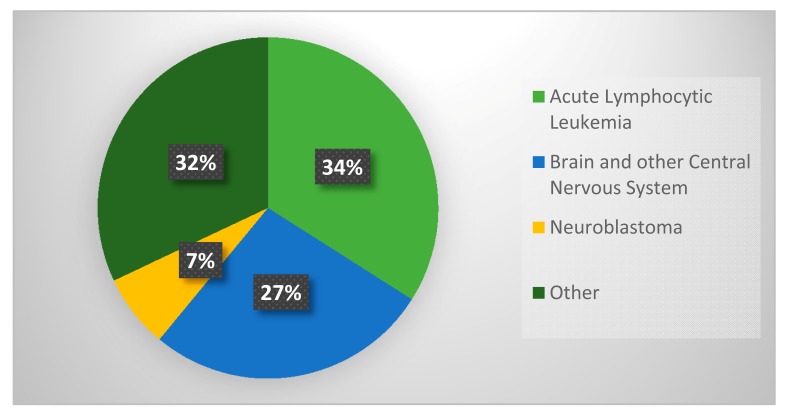
Childhood Cancers.

Similar to progress with infectious diseases, enormous strides have been made in the diagnosis and clinical management of childhood cancers. Although deaths from cancer have decreased by over 70% in the last 40 years, cancer continues to be the leading cause of death associated with childhood disease [5]. This year, it is estimated that over 10,000 new cases of cancer will be diagnosed in children under the age of 15 in the United States, alone [5]. In the field of pediatric oncology, leukemias and brain tumors represent a major crisis and bottleneck for preventable childhood deaths. Figure 2 illustrates the prevalence of the most common forms of pediatric cancer. 

Beyond infectious diseases and cancer, a host of other maladies impact pediatric demographics. These range from acute, life-threatening conditions to chronic diseases which impact quality of life and/or projected lifespan. Common chronic childhood diseases include asthma, diabetes, obesity, and cystic fibrosis, to name a few. 

## 2. Discussion

Despite great progress in the many sub-disciplines of pediatric disease, children are exposed to some of the most painful, debilitating, and deadly diseases. As human health researchers, we have the ability and the obligation to help change this. We encourage the pediatric health research community to come together and share recent findings in an upcoming Special Issue of *Diseases* titled, “Pediatric Diseases.” In this Special Issue, we invite papers related to detection, prevention, diagnosis and treatment of human pediatric diseases. Papers related to molecular mechanisms, epidemiology, immunology, and public health initiatives associated with pediatric diseases are also encouraged. For more information, visit: http://www.mdpi.com/journal/diseases/special_issues/pediatric_diseases.
